# Prevalence of *Toxoplasma gondii* antibodies in domestic donkeys (*Equus asinus*) in Durango, Mexico slaughtered for human consumption

**DOI:** 10.1186/s12917-015-0325-9

**Published:** 2015-01-17

**Authors:** Cosme Alvarado-Esquivel, Domingo Alvarado-Esquivel, Jitender P Dubey

**Affiliations:** Biomedical Research Laboratory, Faculty of Medicine and Nutrition, Juárez University of Durango State, Avenida Universidad S/N, 34000 Durango, Mexico; United States Department of Agriculture, Agricultural Research Service, Beltsville Agricultural Research Center, Animal Parasitic Diseases Laboratory, Building 1001, Beltsville, MD 20705-2350 USA

**Keywords:** *Toxoplasma gondii*, Seroprevalence, Donkeys, Cross-sectional study, Mexico

## Abstract

**Background:**

Nothing is known about *Toxoplasma gondii* prevalence in donkeys in Mexico. Meat from donkey is consumed by humans in Mexico and also exported to other countries. We sought to determine the presence of antibodies against *T. gondii* in 239 domestic donkeys (*Equus asinus*) for slaughter in Durango, Mexico using the modified agglutination test (MAT). Donkeys were sampled in four premises (trade centers) where donkeys were gather for shipment to abattoirs in other Mexican states.

**Results:**

Antibodies to *T. gondii* were found in 26 (10.9%) of 239 donkeys, with MAT titers of 1:25 in 7, 1:50 in 11, 1:100 in 6, and 1:200 in 2. Seropositive donkeys were found in three (75%) of the four gathering premises studied. Seroprevalence in donkeys varied from 0% to 23.1% among gathering premises. The seroprevalence of *T. gondii* infection was comparable among donkeys regardless their age, sex or health status. Seropositivity to *T. gondii* was found in donkeys between 1 to 12 years old. Multivariate analysis showed that seropositivity to *T. gondii* was associated with the gathering premises (OR = 1.58; 95% CI: 1.11-2.24; *P* = 0.009).

**Conclusions:**

This is the first report of *T. gondii* infection in donkeys in Mexico. Results indicate that consumption of undercooked or raw meat from *T. gondii*-infected donkeys is potentially a source of *T. gondii* infection for humans.

## Background

*Toxoplasma gondii* infects warm-blooded animals including equids [1]. Infections with *T. gondii* in equids are of epidemiological importance because their meat and milk are used for human consumption [2]. Infections with *T. gondii* in humans may lead to ocular and nervous system disease [3]. Furthermore, primary *T. gondii* infection in pregnant women may lead to congenital disease with disastrous consequences to the fetus [4]. Immunocompromised individuals may develop a life-threatening toxoplasmosis [5]. Cases of fatal toxoplasmosis due to consumption of imported horsemeat in France have been reported [6]. Also in France, a case of severe pulmonary toxoplasmosis in an immunocompetent man who had consumed imported raw horsemeat was recently reported [7]. These reports in France were epidemiologically linked to consumption of horsemeat from countries in the Americas [6,7]. Therefore, the study of *T. gondii* infection in equids in the Americas is important. We recently reported the seroprevalence of *T. gondii* infection in horses in Durango, Mexico [8]. However, to the best of our knowledge, there is no information concerning *T. gondii* infections in donkeys (*Equus asinus)* in Mexico. Infections with *T. gondii* in equids in Mexico should be of concern for two main reasons: firstly, meat from equids is fraudulently sold as beef for human consumption [9]; and secondly, many equids are road-killed and serve as a meat source for carnivore animals spreading the infection among other animals in the environment [10]. Of note, *T. gondii* DNA has been found in milk and blood from naturally infected donkeys [11]. In addition, viable *T. gondii* was recently isolated from tissues of a donkey in Brazil [12]. In the present study, we sought to determine the seroprevalence of *T. gondii* infection in donkeys for slaughter in Durango, Mexico.

## Methods

### Donkeys studied

Domestic donkeys (n = 239) were sampled from four equids gathering premises (trade centers) in the municipality of Durango, Mexico from July to August 2014 (Table 1). These premises congregate equids for their shipment to abattoirs in other Mexican States. A veterinarian obtained the characteristics of the donkeys by direct observation and from information provided by the owners of the trade centers. A questionnaire was used to obtained the characteristics of the donkeys. The items of the questionnaire included age, sex, breed, health status, and type of feeding of the donkeys, and presence of cats in the trade centers. Most (n = 193) donkeys were apparently healthy and 46 donkeys were defined as ill based on the clinical manifestations observed by a veterinarian: one was malnourished, one had an abdominal mass, and 44 had skin sores. All donkeys were mixed breed and aged 0.2 to 12 years old, 170 (71.1%) were females and 69 (28.9%) males.

**Table 1:** **General data of the 239 donkeys studied and seroprevalence of** ***T. gondii*** **infection**

		**Donkeys tested**	**Seroprevalence of** ***T. gondii*** **infection**		
**Characteristics**		**No.**	**No.**	**%**	***P*** **value**
Age (years)					
	<1	11	0	0	0.25
	1-2	35	3	8.6	
	3-5	100	10	10	
	6-7	33	6	18.2	
	8-10	58	6	10.3	
	>10	2	1	50	
Sex					
	Male	69	4	5.8	0.1
	Female	170	22	12.9	
Health status					
	Ill	46	8	17.4	0.11
	Healthy	193	18	9.3	
Trade center					
	1	170	14	8.2	0.08
	2	5	0	0	
	3	38	6	15.8	
	4	26	6	23.1	

### Ethics statement

All research was performed in accordance with protocols approved by the U.S. Department of Agriculture and the Academic Secretary of the Faculty of Medicine and Nutrition (No. FM-SA-230/14). Efforts were made to minimize any suffering of donkeys during the blood sampling. A verbal consent was obtained from the owners of the donkeys.

### Serological examination

Blood was drawn from donkeys by direct jugular venipuncture. Sera were obtained by blood centrifugation and stored at −20°C until tested. Donkeys’ sera were tested for *T. gondii* antibodies using 2-fold serial dilutions from 1:25 to 1:3,200 with the modified agglutination test (MAT) as described by Dubey and Desmonts [13]. A titer of 1:25 was used as cut off for seropositivity in MAT.

### Statistical analysis

Statistical analysis was performed using the software Epi Info 7 (Centers for Disease Control and Prevention: http://wwwn.cdc.gov/epiinfo/) and SPSS version 15.0 (SPSS Inc. Chicago, Illinois). We used the Pearson’s chi-squared test or, when indicated, the Fisher exact test for comparison of the frequencies among groups. We assessed the association between the donkeys’ characteristics and *T. gondii* seropositivity by multivariable analysis (logistic regression analysis with the Enter method). Independent variables included in the multivariate analysis were only those with *P* < 0.15 obtained in the bivariate analysis. The dependent variable was *T. gondii* seropositivity by MAT for an individual donkey. Independent variables included in the multivariable analysis were sex, health status and gathering premise (trade center). We used the Hosmer-Lemeshow goodness of fit test to assess the fitness of the regression model. Statistical significance was set at a *P*-value of < 0.05.

## Results

Antibodies to *T. gondii* were found in 26 (10.9%) of 239 donkeys, with titers of 1:25 in 7, 1:50 in 11, 1:100 in 6, and 1:200 in 2. Seropositive donkeys were found in three (75%) of the four gathering premises studied (Table 1). Seroprevalence in donkeys varied from 0% to 23.1% among gathering premises *(P* = 0.08). The seroprevalence of *T. gondii* infection was comparable among donkeys regardless their age. The youngest donkey with *T. gondii* antibodies was 1 year old and the oldest donkey with *T. gondii* antibodies was 12 years old. Further analysis of donkeys’ characteristics by age groups did not show an association with *T. gondii* seropositivity. According to the information provided by the donkeys’ owners, all donkeys pastured. No cats were seen in the gathering premises. The variables sex, ill status, and trade center showed *P* values lower than 0.15 and were included in the regression analysis. Age was not included in the regression analysis because it did not show a correlation with *T. gondii* seropositivity by bivariate analysis. Multivariate analysis showed that seropositivity to *T. gondii* was associated with the trade centers (OR = 1.58; 95% CI: 1.11-2.24; *P* = 0.009) (Table 2). Other characteristics of donkeys including sex and ill status were not associated with *T. gondii* seropositivity by multivariate analysis. The result of the Hosmer-Lemeshow test was 5 (*P* = 0.46), indicating an acceptable fit of our regression model.

**Table 2:** **Multivariate analysis of selected characteristics of donkeys**

**Characteristic**	***P*** **value**	**Odds ratio**	**95% confidence interval**
Sex	0.11	2.51	0.80-7.86
Ill status	0.05	2.53	0.97-6.55
Trade center	0.009	1.58	1.11-2.24

## Discussion

The 10.9% seroprevalence of *T. gondii* we found is of epidemiological importance since donkeys sampled were for human consumption either in Mexico or abroad. In other surveys from Brazil, Egypt, Italy, Spain, Turkey, and China antibodies to *T. gondii* were found in 1.5% to 65.6% of donkeys surveyed but comparisons are difficult because of different serological tests used [2,12,14-20]. However, using the same MAT and same cut off titer, 6.4% of 375 donkeys from the USA were seropositive [21]. The seroprevalence in donkeys in the present study is higher than the 6.1% seroprevalence of *T. gondii* infection reported in horses in Durango, Mexico using the same MAT [8]. However, comparison of seroprevalences in horses and donkeys in Durango should be interpreted with care because of a number of differences among the studies. Apart from the difference in equids species, differences in the characteristics of the raising environment among the studies exists. In the present work, donkeys pastured in rural environment whereas horses included in the past study were fed both in stalls and in the fields and included both rural and urban animals. It is likely that horses received a better care in hygiene of drinking water and food than donkeys for slaughter. Horses had probably good care from their owners because many horses were used for working purposes. Instead, donkeys for slaughter are usually unwanted animals, and many of them are ill.

In the present study seropositivity to *T. gondii* was associated with trade center. This fact may help abattoirs for selecting trade centers for the purchase of donkeys with low seroprevalence of *T. gondii* infection. It is unclear why donkeys of some trade centers had higher seroprevalences than those in other trade centers. The traders buy all donkeys regardless their age, sex or health status. Seropositivity to *T. gondii* was not associated with age, sex, or health status of donkeys. All donkeys were fed in the fields, and cats were not seen in the gathering premises. Therefore, these factors could not be analyzed for their contributing role in *T. gondii* exposure in the donkeys studied. The difference in seroprevalence of *T. gondii* infection among the trade centers might be related with past contact with cats in the origin places of the donkeys. However, contact with cats could not be traced back to the origin places because the owners of the gathering premises had a very limited information about the history of the donkeys bought.

We found seropositive donkeys in three of four gathering premises. However, the premise without seropositive donkeys provide us only five donkeys for sampling. This small sample size cannot rule out seropositivity in donkeys in such premise. Anyway, results suggest a wide dissemination of *T. gondii* infection in donkeys for slaughter in Durango, Mexico. All *T. gondii* seropositive donkeys were aged from one to twelve years old indicating postnatal infections. However, the number of donkeys aged less than 1 year old was small (n = 11) to exclude *T. gondii* infections in this youngest age group. The fact that DNA of *T. gondii* has been found in blood and milk of *T. gondii*-infected donkeys [9] raises concern for the risk of *T. gondii* infection to humans by consuming meat or milk from donkeys.

## Conclusions

We conclude that seropositivity to *T. gondii* is common among donkeys for slaughter in Durango, Mexico. This is the first seroprevalence study of *T. gondii* infection in donkeys in Mexico. Consumption of raw or undercooked meat from *T. gondii*-infected donkeys may represent a risk of infection for humans.

## Abbreviations

MAT: Modified agglutination test
OR: Odds ratio
CI: Confidence interval
